# PReS-FINAL-2004: Musculoskeletal pain in schoolchildren across puberty: a 3-year follow-up study

**DOI:** 10.1186/1546-0096-11-S2-O7

**Published:** 2013-12-05

**Authors:** F Sperotto, S Brachi, G Cuffaro, M Seguso, F Vittadello, F Zulian

**Affiliations:** 1 Department of Pediatrics, University of Padua, Padua, Italy; 2 Department of Laboratory Medicine, University of Padua, Padua, Italy

## Introduction

Chronic Musculoskeletal Pain (MSP) in children can be due to various non-inflammatory conditions, such as the benign joint hypermobility syndrome (BJHS) or idiopathic MSP (IMSP). MSP heavily influences patient's quality of life and is often misdiagnosed or included in the vast category of unspecific MSP. A careful differential diagnosis and knowledge of possible risk factors are needed to properly approach a child/adolescent with osteoarticular symptoms.

## Objectives

Aim of the study was to evaluate the persistence of osteoarticular symptoms in a cohort of schoolchildren with MSP followed for three years and to analyze the main risk factors for it, with particular attention to the role of the pubertal stage. We also evaluated the immunological profile and its relation with MSP and puberty.

## Methods

The participants were selected among an initial cohort of 88 schoolchildren with MSP, aged 11-16 years, followed for three years. Chronic MSP was defined as continuous or recurrent pain lasting more than 3 months interfering with daily life activities, according to the International Association for the Study of Pain, and it was researched in the previous 6 months. As at baseline, subjects with past of present sign of any neurological, skeletal, metabolic or autoimmune conditions were excluded. All children underwent a general and rheumatologic examination focused on the presence of generalized joint hypermobility, identified by Beighton score ≥4/9, the body mass index and the pubertal stage. Finally, a laboratory test was performed to determine the presence of ANA, ENA and anti-dsDNA.

## Results

Seventy subject entered the study: 38 (54.3%) still presented MSP, including 27% with BJHS and 27% with IMSP. Main symptoms were lower limbs arthralgia and myalgia. MSP persisted more in females than in males (p = 0.038) and in pubertal rather than pre-pubertal subjects (p = 0.022). In particular, the pre-pubertal patients recovered significantly more frequently from BJHS (p = 0.004) and IMSP (p = 0.016) than the pubertal ones. Gender did not influence the distribution of MSP according to pubertal stage. ANA (titer ≥1:80) was found in 42.9%; none of the subjects resulted positive at ENA or anti-dsDNA tests. No significant association between ANA-positivity and MSP or BJHS was found.

## Conclusion

Pre-pubertal patients have a higher probability of recovering from MSP than the pubertal ones; vice versa pubertal subjects are at high risk for suffering from MSP during early adulthood, especially females with BJHS. The prevalence of ANA-positivity in this cohort was particularly high but was not significantly associated with BJHS or IMSP. These findings clearly suggest that female gender, BJHS and pubertal stage are important risk factors for persistence of MSP. Further studies are needed to evaluate the natural history of MSP towards adulthood and the role of ANA in the pubertal age.

## Disclosure of interest

None declared.

